# Three new species, and new distributional data, of *Haltichella* (Hymenoptera, Chalcididae) from China

**DOI:** 10.3897/zookeys.1060.70427

**Published:** 2021-09-15

**Authors:** Zi-Tong Wang, Cheng-De Li

**Affiliations:** 1 School of Forestry, Northeast Forestry University, Harbin, 150040, China Northeast Forestry University Harbin China

**Keywords:** Chalcidoidea, chalcid wasps, Haltichellini, Haltichellinae, identification key, taxonomy

## Abstract

Five species of *Haltichella* (Hymenoptera, Chalcididae) from China are reviewed, including three new species, *H.bimaculata* Wang & Li, **sp. nov.**, *H.bomiana* Wang & Li, **sp. nov.**, *H.strigata* Wang & Li, **sp. nov.***Haltichellaclavicornis* (Ashmead) is newly recorded from China and *H.nipponensis* Habu is newly recorded from Heilongjiang, Shanxi, Shandong, Xizang, Guangdong and Yunnan Provinces in China. A key to the Chinese species of the *Haltichella* is provided.

## Introduction

The genus *Haltichella* Spinola, 1811 (Chalcididae, Haltichellinae, Haltichellini) currently contains 35 valid species worldwide (Noyes 2019), but only four species were recorded from China (Walker 1862; Narendran 1989). Members of the genus possess a cosmopolitan distribution and are primarily parasitoids of Lepidoptera (Bucculatricidae, Gelechiidae, Momphidae, Notodontidae, Oecophoridae, Pyralidae, Tortricidae) and Hymenoptera (Braconidae, Ichneumonidae) (Narendran and van Achterberg 2016; Noyes 2019). Here we review five species of the genus *Haltichella* from China, including three new species, and a new report of *H.clavicornis* Habu for China. Distributional records of *H.nipponensis* and a key to species of *Haltichella* occurring in China are also provided.

## Materials and methods

Specimens were collected by using sweep nets, yellow pan traps and Malaise traps, and glued to triangle cards. Photographs were taken with a digital CCD camera attached to an AOSVI Hk830 microscope. Relative measurements and total body lengths were measured using an eye-piece reticle in the ocular of the microscope. All the specimens examined were deposited in the insect collections at Northeast Forestry University (NEFU), Harbin, China. In addition, literature used to identify this genus mainly include the keys and original descriptions of Narendran (1989), Narendran and van Achterberg (2016), Roy and Farooqi (1984). Terminology follows that of the Hymenoptera Anatomy Consortium (2021). The following abbreviations are used for the repositories:

**IARI**Indian Agricultural Research Institute, New Delhi, India;

**NEFU** Northeast Forestry University, Harbin, China;

**NIAS** National Institute of Agricultural Sciences, Tokyo, Japan;

**USNM**United States National Museum of Natural History, Washington D.C., USA.

The following abbreviations are used in the text:

**Fu1–7** funiculars 1–7;

**OOL** oculo-ocellar distance, minimum distance between a posterior ocellus and eye;

**POL** postocellar distance, the distance between both posterior ocelli;

**Gt1–2** tergites 1–2 of metasoma;

**YPT** yellow pan trap.

## Results

### Key to Chinese species of *Haltichella Spinola*

**Table:** 

1	Apex of mesoscutellum not emarginate and without any distinct tooth	**2**
–	Apex of mesoscutellum emarginate forming two distinct teeth (Figs 1H, 2I)	**3**
2	Forewing with two blackish bands, marginal vein 0.25× as long as submarginal vein; metasoma fusiform, a little longer but not narrower than mesosoma	***H.sulcator* Walker**
–	Forewing without blackish bands, marginal vein half as long as submarginal vein; metasoma elongate-oval, a little narrower but hardly longer than mesosoma	***H.finator* Walker**
3	Metasoma with more than two basal carinae on Gt1 (Figs 5B, 6D) or with some longitudinal striae between two carinae (Fig. 3H)	**4**
–	Metasoma with only two basal carinae on Gt1	**5**
4	Mesoscutellum with a median longitudinal fovea; apex of mesoscutellum with two diverging and short teeth (Fig. 4E); eye without obvious setae (Fig. 4B, 5D)	***H.clavicornis* (Ashmead)**
–	Mesoscutellum without a median longitudinal fovea; apex of mesoscutellum with two diverging and longer teeth (Fig. 3I); eye with dense and long setae (Fig. 3D)	***H.strigata* Wang & Li, sp. nov.**
5	Forewing with two brown patches (Fig. 1E); scrobe reaching anterior ocellus	**6**
–	Forewing at most with one patch; scrobe not reaching anterior ocellus (Figs 2B, 4B)	**7**
6	Postmarginal vein absent (Fig. 1E); submedian carinae not obvious and median area of propodeum irregularly rugose (Fig. 1F); mesoscutellum as long as broad	***H.bimaculata* Wang & Li, sp. nov.**
–	Postmarginal vein present and shorter than stigmal vein (Fig. 7C); submedian carinae distinct and parallel (Fig. 7F); mesoscutellum longer than broad (Fig. 7I)	***H.nipponensis* Habu**
7	First tooth of metafemural comb of teeth prominent; postmarginal vein longer than marginal vein	***H.variicolor* Masi**
–	First tooth of metafemural comb of teeth not prominent (Fig. 2G); postmarginal vein about 2/3 the length of marginal vein (Fig. 2E)	***H.bomiana* Wang & Li, sp. nov.**

#### Haltichella bimaculata

Taxon classificationAnimaliaHymenopteraChalcididae

Wang & Li sp. nov.

43580AAF-3CCE-5668-9419-3C710ECF7B4F

http://zoobank.org/9EAF85B7-E3C5-4A2C-AF4F-B31E6DE62ECF

Figure 1

##### Type material.

***Holotype*,** ♀ (NEFU), China, Henan Province, Xinyang City, 17–18.V.2012, YPT, Guo-Hao Zu, Jiang Liu. ***Paratypes*** (NEFU): 1 ♀, China: Yunnan Province, Huanglianshan Nature Reserve, 27–28.VII.2018, YPT, Jun Wu, Ming-Rui Li.

##### Diagnosis.

**Figure 1.**
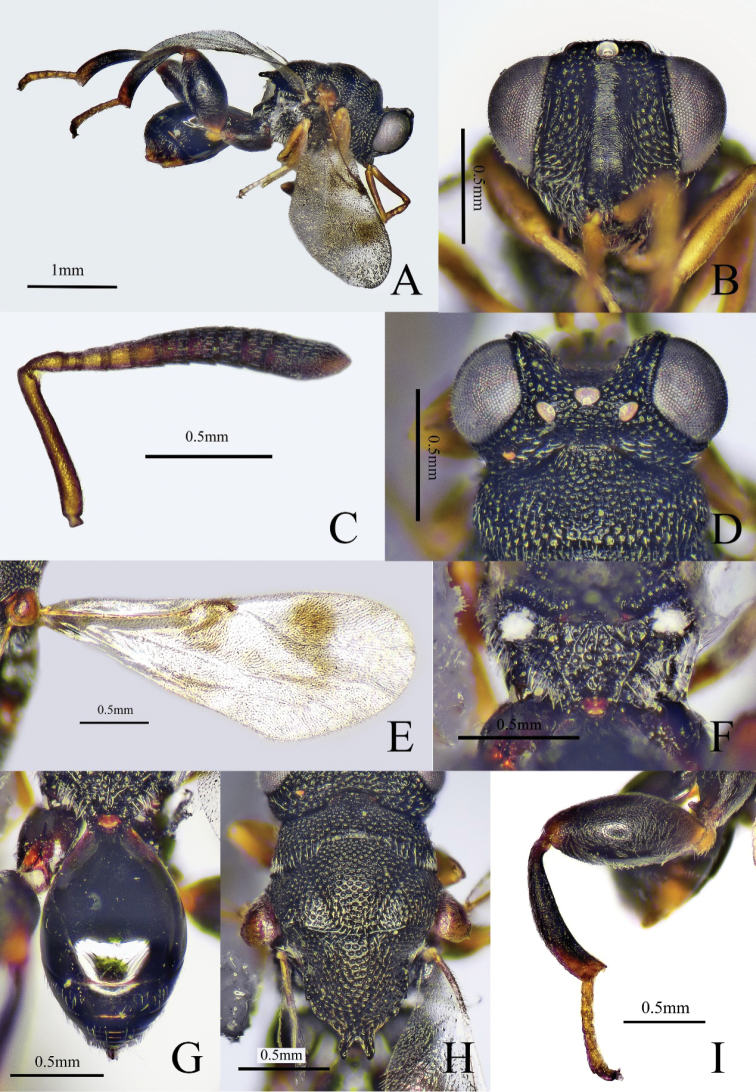
*Haltichellabimaculata* sp. nov. (holotype female) **A** habitus, lateral view **B** head, front view **C** antenna **D** head and part of mesosoma, dorsal view **E** forewing **F** propodeum **G** metasoma **H** head and mesosoma, dorsal view **I** hind leg.

Body black (Fig. 1A), scape to Fu3 yellowish brown, Fu4–7 and club dark brown (Fig. 1C), fore wing cinereous with two brown patches (Fig. 1E); scape (Fig. 1C) approximately half as long as remaining antennomeres combined, pedicel 1.5× as long as Fu1, Fu1–7 gradually increases in breadth and Fu4–7 gradually decreases in length distad; mesoscutellum (Fig. 1H) as long as broad, apex with two teeth, distance between outer margins of the two teeth about 1.4× as long as individual length of teeth; propodeum (Fig. 1F) with irregularly rugose in middle area; postmarginal vein (Fig. 1E) absent, marginal vein 3× as long as stigmal vein; metasoma oval; Gt1 occupying 0.7× length of metasoma (Fig. 1H).

##### Description.

**Female** (holotype). Body length 2.9 mm, mostly black (Fig. 1A), with dense punctures and white pubescence; antenna (Fig. 1C) with scape to Fu3 yellowish brown, Fu4–7 and club dark brown; eye and ocelli silvery gray (Fig. 1D); tegula testaceous; fore and mid legs yellowish brown; metacoxa black with apical reddish brown, metatrochanter yellowish brown, metafemur black with base yellowish brown to reddish brown, apex reddish brown, metatibia black with base slightly reddish brown, apex yellowish brown, metatarsus yellowish brown; fore wing (Fig. 1E) cinereous with two brown patches, one adjoining marginal vein and another near posterior margin of wing, venation brown.

***Head*** (Fig. 1B, D), with coarsely rugose punctures, except in scrobal area, 1.2× as wide as long in frontal view; scrobe reaching anterior ocellus, finely striate; preorbital carinae distinct; POL 4.3× as long as OOL; antenna (Fig. 1C) clavate; scape approximately a half of remaining antennomeres combined; pedicel triangular, longer than broad; anellus quadrate, looks like a funicular segment; Fu1–2 subquadrate, Fu1 shorter than pedicel, 0.9× as broad as long; Fu3–7 broader than long; Fu1–7 gradually increases in breadth and Fu4–7 gradually decreases in length distad, Fu7 1.5× as broad as long; club coniform, 2× as long as maximum width, 3× as long as and about as broad as the preceding segment.

***Mesosoma*** (Fig. 1H), punctures on mesoscutum and mesoscutellum smaller than on head; mesoscutellum as long as broad, apex with two teeth, distance between outer margins of the two teeth about 1.4× as long as individual length of teeth; outer margins of the two teeth approximately parallel, inner margins of the two teeth meet at an acute angle; propodeum (Fig. 1F) irregularly rugose in middle area. Forewing (Fig. 1E) 2.5× as long as broad; submarginal vein 4.5× as long as marginal vein, marginal vein 3× as long as stigmal vein, postmarginal vein absent. Metacoxa with coxal tooth on baso-dorsal side; metafemur (Fig. 1I) 2.2× as long as broad, with a row of comb of teeth but without forming any lobes.

***Metasoma*** (Fig. 1G) oval, 1.6× as long as broad in dorsal view, surface smooth; Gt1 longest, occupying 0.7× length of metasoma, with two short carinae at base; Gt2–6 with sparse microsculptured and white pubescence on lateral sides.

**Male.** Unknown.

##### Hosts.

Unknown.

##### Distribution.

China (Hennan, Yunnan).

##### Etymology.

Latin: *bi* = two; *macula* = stain, blemish; and refers to the two brown patches on the forewing.

##### Comments.

*Haltichellabimaculata* sp. nov. is similar to *H.nipponensis* Habu, 1960 in having similar body colouration and shape of the antenna and similar shape of the metafemur, but can be separated from the latter by the following characters. *Haltichellabimaculata* has the postmarginal vein of the forewing absent (vs present and shorter than the stigmal vein in *H.nipponensis*); median area of the propodeum irregularly rugose and the submedian carinae not obvious (vs less sculptured and the carinae distinct and parallel); the mesoscutellum as long as broad (vs longer than broad).

#### Haltichella bomiana

Taxon classificationAnimaliaHymenopteraChalcididae

Wang & Li sp. nov.

71116FBE-EBDD-51EE-BCB6-5FE18FE01FA4

http://zoobank.org/676C10E6-E55A-42E3-8C39-8273B21BC3A8

Figure 2

##### Type material.

***Holotype*,** ♂ (NEFU), China, Xizang Province, Bomi County, Shuangyu Village, 8.VIII.2017, sweeping, Hui-Lin Han. ***Paratypes*** (NEFU): 2 ♂, same data as holotype.

##### Diagnosis.

**Figure 2.**
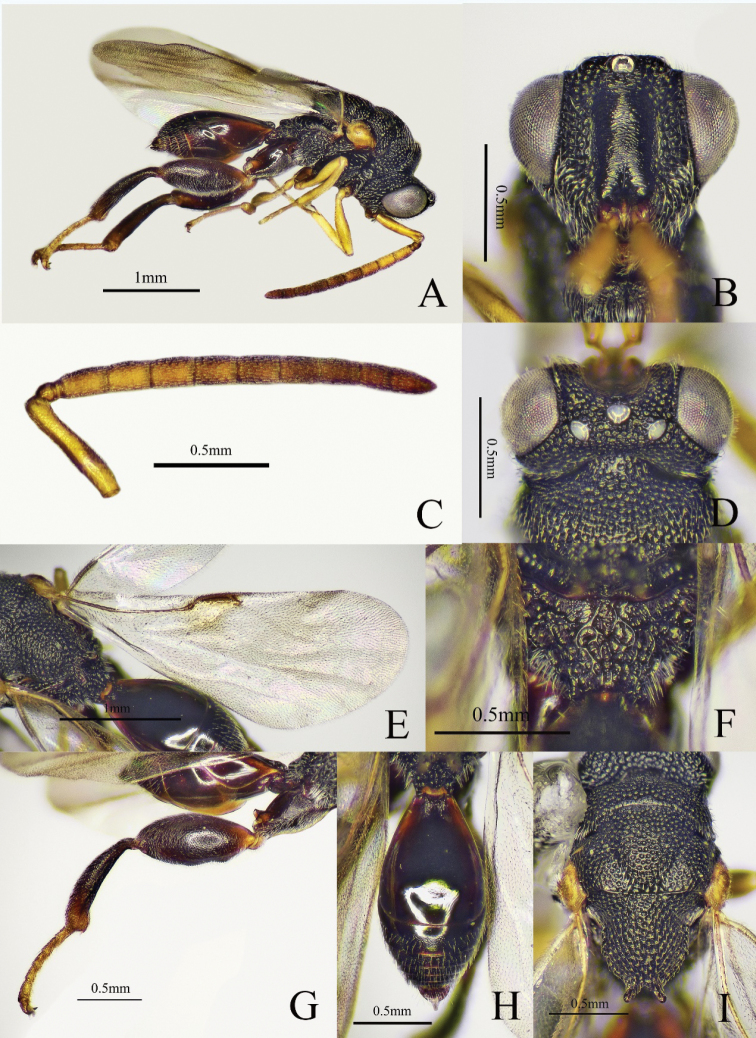
*Haltichellabomiana* sp. nov. (holotype male) **A** habitus, lateral view **B** head, front view **C** antenna **D** head and part of mesosoma, dorsal view **E** forewing **F** propodeum **G** hind leg **H** metasoma **I** mesosoma, dorsal view.

Body black (Fig. 2A), antenna with scape yellowish brown and flagellum yellowish brown to brown; fore and mid legs yellowish brown; scrobe not reaching anterior ocellus (Fig. 2B); antenna (Fig. 2C) slender, scape longer than pedicel to Fu2 combined; all the funicular segments longer than broad; mesoscutellum apically with two diverging teeth (Fig. 2I); submedian carinae of propodeum indicated, distinct on posterior half (Fig. 2F); fore wing (Fig. 2E) largely hyaline with small brown patch adjoining marginal vein; postmarginal vein slightly longer than stigmal vein; metasoma (Fig. 2H) fusiform, Gt1 occupying 0.6× length of metasoma, Gt2 with the basal half smooth and distal half with microsculptured and white pubescence.

##### Description.

**Male** (holotype). Body length 2.9 mm, mostly black (Fig. 2A), head and mesosoma with dense punctures and white pubescence; antenna with scape yellowish brown and flagellum yellowish brown to brown; eye and ocelli silvery gray (Fig. 2D); tegula yellowish brown; fore and mid legs yellowish brown; metacoxa black, metatrochanter yellowish brown, metafemur black with base yellowish brown to reddish brown, metatibia black with apex yellowish brown, metatarsus yellowish brown; fore wing largely hyaline with small brown patch adjoining marginal vein and venation brownish.

***Head*** (Fig. 2B, D) with coarsely rugose punctures, 1.2× as wide as long in frontal view; scrobe slightly wider, not reaching anterior ocellus, finely striate; preorbital carinae distinct; POL 5× as long as OOL; antenna (Fig. 2C) slender; scape longer than pedicel to Fu2 combined; pedicel small; anellus short and transverse; Fu1 longest, 1.7× as long as broad; Fu2–7 approximately equal in length, all the funicular segments longer than broad; club 2-segmented and slender, 3.2× as long as maximum width, 2× as long as and as broad as the preceding segment.

***Mesosoma*** (Fig. 2I) with dense punctures and white pubescence; apex of mesoscutellum with two diverging teeth, inner margins of the two teeth at a right angle; submedian carinae of propodeum (Fig. 2F) indicated, distinct on posterior half. Fore wing hyaline (Fig. 2E), forewing 2.6× as long as broad; submarginal vein 4× as long as marginal vein, marginal vein 1.6× as long as postmarginal vein, postmarginal vein slightly longer than stigmal vein. Metafemur and metatibia with long and white pubescence (Fig. 2G); metacoxa with coxal tooth on baso-dorsal side; metafemur 2.3× as long as broad, with a row of comb of teeth.

***Metasoma*** (Fig. 2H) fusiform, 2× as long as broad in dorsal view; Gt1 dorsum smooth and shiny, occupying 0.6× length of metasoma, with two longitudinal carinae at base; Gt2 with basal half smooth and distal half with microsculptured and white pubescence; dorsal surface of Gt2–6 with sparse microsculptured and white pubescence.

**Female.** Unknown.

##### Hosts.

Unknown.

##### Distribution.

China (Xizang).

##### Etymology.

The specific name is derived from the name of the collection locality of the holotype.

##### Comments.

The new species is similar to *H.variicolor* Masi, 1929 in having similar colouration and shape of the antenna and similar shape of the scrobe, but can be separated from the latter by the following characters. The new species has the marginal vein 1.6× as long as the postmarginal vein (vs postmarginal vein longer than marginal vein in *H.variicolor*) and metafemur without prominent first tooth (vs first tooth present and prominent).

The new species is characterized by two diverging teeth of the mesoscutellum, a unique propodeum and the scrobe not reaching the anterior ocellus. A female holotype would be preferable but we failed to collect female specimens. However, most likely the female will share at least part of the differences listed for the male holotype.

#### Haltichella strigata

Taxon classificationAnimaliaHymenopteraChalcididae

Wang & Li sp. nov.

898F02E1-2C2C-5CED-A3CB-FC97FC3D1D7B

http://zoobank.org/A1242FA3-10BA-4CD1-BACA-7EDBEB6AE821

Figure 3

##### Type material.

***Holotype*,** ♂ (NEFU), China, Heilongjiang Province, Harbin City, Maoershan Town, 19.VII.2014, sweeping, Hai-Feng Bai. ***Paratypes*** (NEFU): 1 ♂, China: Heilongjiang Province, Yichun City, Liangshui National Nature Reserve, 11.VII.2013, sweeping, Hui Geng, Yang Peng, Si-Zhu Liu, Guo-Hao Zu; 1 ♂,China: Heilongjiang Province, Yichun City, Liangshui National Nature Reserve, 1.VIII.2015, sweeping, Xing-Yue Jin, Si-Zhu Liu, Xin-Yu Zhang; 1 ♂, China: Heilongjiang Province, Yichun City, Fenglin National Nature Reserve, 15.VII.2011, sweeping, Jun-Chao Wang.

##### Diagnosis.

**Figure 3.**
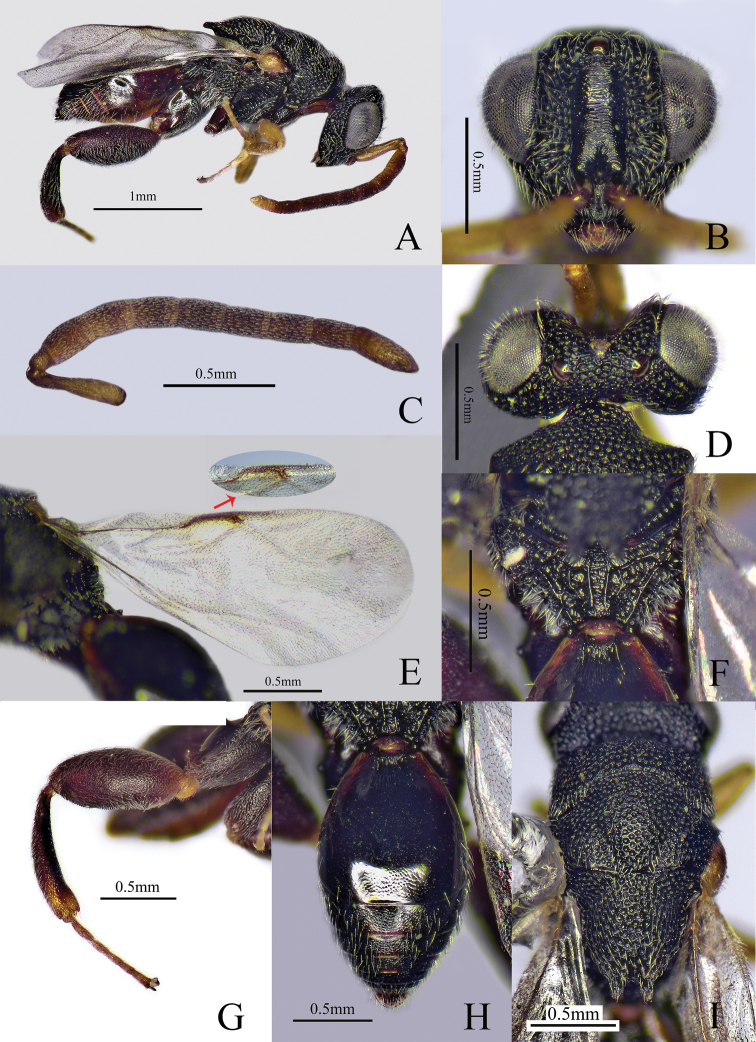
*Haltichellastrigata* sp. nov. (holotype male) **A** habitus, lateral view **B** head, front view **C** antenna **D** head, dorsal view **E** forewing **F** propodeum **G** hind leg **H** metasoma **I** mesosoma, dorsal view.

Body mostly black (Fig. 3A), antenna with scape yellowish brown, funicle yellowish brown to brown and club yellowish brown; fore and mid legs yellowish brown, metafemur reddish brown; eye (Fig. 3D) with dense and long setae; scape shorter than pedicel to Fu2 combined; Fu1 longest; all the funicular segments longer than broad; postmarginal vein shorter than marginal vein and 3.5× as long as stigmal vein (Fig. 3E); Gt1 with two longitudinal carinae at base, between them with some longitudinal striae (Fig. 3H).

##### Description.

**Male** (holotype). Body length 2.9 mm, mostly black (Fig. 3A). Head and mesosoma with dense punctures and white pubescence; antenna (Fig. 3C) with scape yellowish brown, funicle yellowish brown to brown and club yellowish brown; eye silvery gray and ocelli brownish (Fig. 3D); tegula yellowish brown; fore and mid legs yellowish brown; hind leg mostly reddish brown except yellowish brown tarsus, distal apex of tibia yellowish brown; fore wing hyaline, venation brownish.

***Head*** (Fig. 3B, D) with long, white pubescence, 1.2× as wide as long in frontal view; eye with long, white setae; scrobe not reaching anterior ocellus, finely striate; preorbital carinae distinct and not reaching behind anterior ocellus; POL 5× as long as OOL; antenna (Fig. 3C) with scape shorter than pedicel to Fu2 combined; pedicel small; Fu1 longest, 1.7× as long as broad; all the funicular segments longer than broad; Fu2–7 subequal in length; club 2-segmented, 2.6× as long as maximum width, 1.8× as long as and about as broad as the preceding segment.

***Mesosoma*** (Fig. 3I) with dense punctures and white pubescence, apex of mesoscutellum with two teeth, pubescence on mesoscutellum longer than that on pronotum and mesoscutum; propodeum (Fig. 3F) with submedian carinae distinct and parallel, between them with some transverse and fine striations. Fore wing hyaline (Fig. 3E), 2.4× as long as broad; postmarginal vein shorter than marginal vein and 3.5× as long as stigmal vein. Metafemur (Fig. 3G) and metatibia with long and white pubescence; metacoxa with coxal tooth on baso-dorsal side; metafemur 2.2× as long as broad, with a row of comb of teeth but without forming any lobes.

***Metasoma*** (Fig. 3H) oblong, 1.6× as long as broad in dorsal view; Gt1 longest, occupying 0.6× length of metasoma with two longitudinal carinae at base, between them with some longitudinal striae, with white pubescence on lateral sides; dorsal surface of Gt2–6 with microsculptured and white pubescence.

**Female.** Unknown.

##### Hosts.

Unknown.

##### Distribution.

China (Heilongjiang).

##### Variation.

Two paratypes differ from the holotype by having black eyes, but no other significant differences were found in the available material.

##### Etymology.

Latin: *stria* = furrow, line; and refers to the longitudinal striae between two longitudinal carinae of T1.

##### Comments.

*Haltichellastrigata* sp. nov. is similar to *H.achterbergi* Narendran, 1989 in having a similar shape of the antenna and the metasoma, but can be separated from the latter by the following combination of characters. The new species has the fore wing hyaline (vs partly infuscated in *H.achterbergi*); Gt1 with some striae between the two longitudinal carinae (vs absent); postmarginal vein shorter than the marginal vein (vs longer); dorsal surface of Gt 2–6 with white pubescence (vs glabrous and polished on the dorsal side medially).

Compared with other species of this genus, the new species differs by having dense and long setae on its eyes (Fig. 3D) and Gt1 with some striae between the two longitudinal carinae. A female holotype would be preferable but we failed to collect female specimens. However, most likely the female will share at least part of the differences listed for the male holotype.

##### Distribution.

New distributional records for China.

#### Haltichella clavicornis

Taxon classificationAnimaliaHymenopteraChalcididae

(Ashmead, 1904)

AE0C50C7-D82C-5615-96B1-3286118BB3A8

Figures 4 , 5 , 6

Stomatoceras clavicornis Ashmead, 1904: 148; holotype ♂, USNM, Japan, not examined.
Haltichella clavicornis : Habu 1960: 241.
Haltichella macroclava Roy & Farooqi, 1984: 27; holotype ♀, IARI, India, not examined. Synonymised with Haltichellaclavicornis by Narendran 1989: 152–153.

##### Material.

(NEFU). 1 ♀, **China**: Henan Province, Xinyang City, Wusheling, 7–9. VIII. 2015, YPT, Yan Gao, Hui Geng, Zhi-Guang Wu; 1 ♀ 2 ♂, China: Hainan Province, Haikou City, Haida Base, 27–29.IV.2019, YPT, Yu-Ting Jiang; 1 ♀, id., but Pinnacle Ridge, 17–19.V.2021, Gang Fu, Ming-Rui Li; 1 ♀, China: Yunnan Province, Yuanjiang County, 26–28.XI.2020, YPT, Jun-Jie Fan, Ming-Rui Li, Gang Fu, Jun Wu; 1 ♀, id., but Mengla Town, Mengla County, 17–18. XI. 2020.

##### Diagnosis.

**Figure 4.**
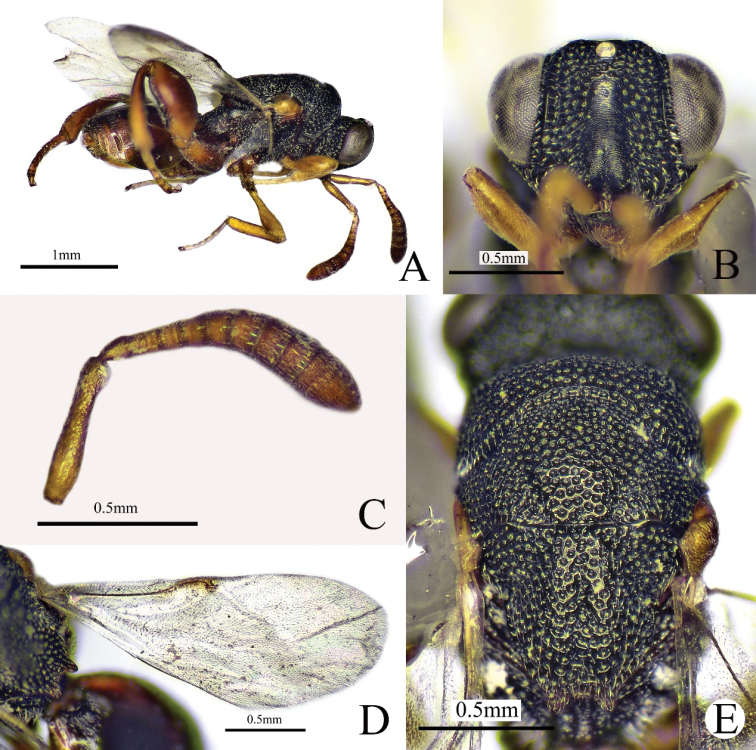
*Haltichellaclavicornis*, (female) **A** habitus, lateral view **B** head, front view **C** antenna **D** forewing **E** mesosoma, dorsal view.

**Figure 5.**
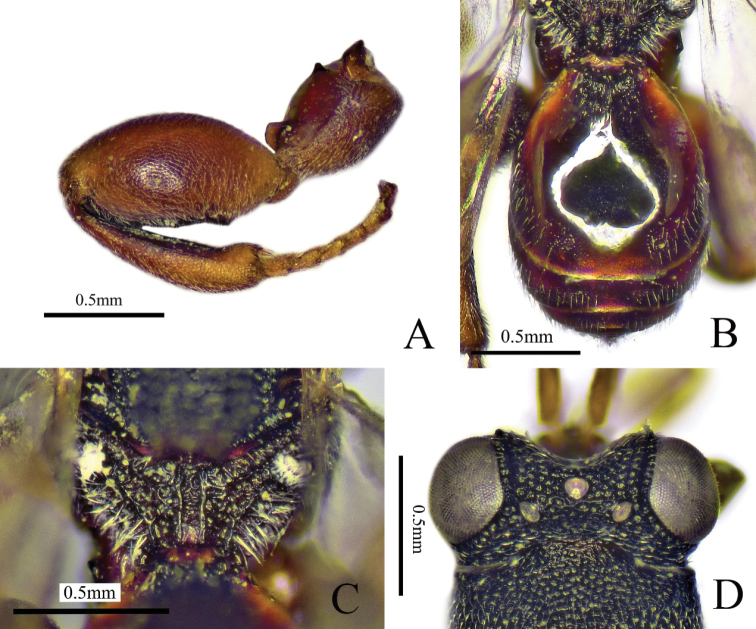
*Haltichellaclavicornis*, (female) **A** hind leg **B** metasoma **C** propodeum **D** head and mesosoma, dorsal view.

**Female.** Body length 2.8–3.1 mm, mostly black (Fig. 4A); antenna with scape and pedicel yellowish brown, flagellum brown to dark brown (Fig. 4C); eye and ocelli silvery gray (Fig. 5D); tegula yellowish brown; fore and mid legs yellowish brown; hind leg (Fig. 5A) and metasoma reddish brown (Fig. 5B); fore wing hyaline (Fig. 4D), venation brownish. Head 1.3× as wide as long in frontal view (Figs 4B, 5D); scrobe not reaching anterior ocellus (Fig. 4B); POL 4.3× as long as OOL; antenna (Fig. 4C) distinctly clavate; pedicel longer than Fu1, flagellum increases in breadth distad; mesoscutellum (Fig. 4E) flat with a median longitudinal fovea; apex of mesoscutellum with two short diverging and short teeth; submedian carinae of propodeum parallel (Fig. 5C); postmarginal vein (Fig. 4D) shorter than stigmal vein. Metasoma (Fig. 5B) oval, Gt1 occupying 0.75× length of metasoma, with three longitudinal carinae at base.

**Figure 6.**
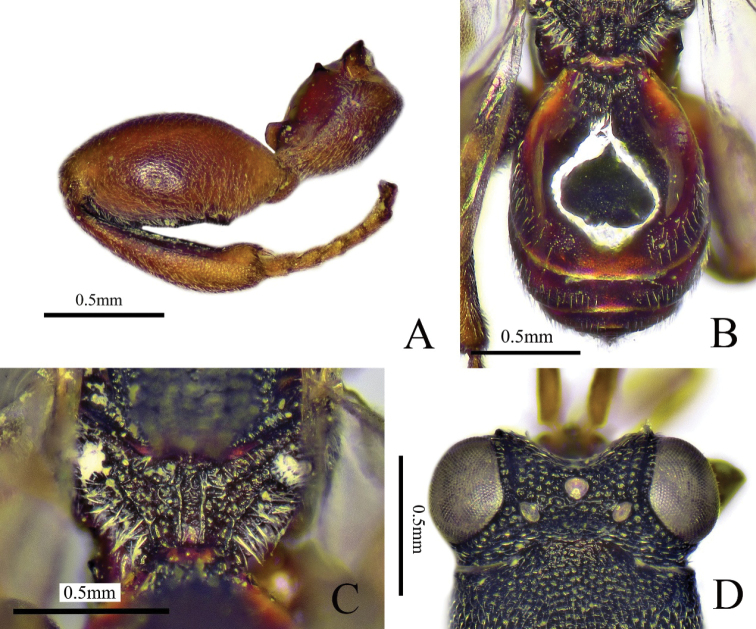
*Haltichellaclavicornis*, (male) **A** habitus, lateral view **B** antenna **C** forewing **D** metasoma.

**Male.** Body length 2.8–2.9 mm. Scape and pedicel brown, flagellum brownish black (Fig. 6B); hind leg black except brown tarsus, extreme apex of femur brown, distal apex of tibia brown; metasoma black (Fig. 6A); anellus not obvious; Fu1–7 subquadrate; club 2× as long as and as broad as the preceding segment (Fig. 6B). Gt1 occupying 0.6× length of metasoma (Fig. 6D), distal half of Gt1 with microsculpture. Other features similar to female.

##### Hosts.

Unknown.

##### Distribution.

China (Henan [new record], Hainan [new record], Yunnan [new record]), Japan, Laos, India, Malaysia, Vietnam, Nepal, Philippines (Narendran 1989; Narendran and van Achterberg 2016).

##### Comments.

Our specimens agree well with the original description except for the colour of the antenna and metasoma. This is the first record from Henan, Hainan and Yunnan Provinces, China.

#### Haltichella nipponensis

Taxon classificationAnimaliaHymenopteraChalcididae

Habu, 1960

4CF3F3AF-E2D4-52FD-B051-0F2C81777472

Figure 7

Haltichella nipponensis Habu, 1960: 245; holotype ♀, NIAS, Japan, not examined.

##### Material.

(NEFU). 2 ♀, **China**: Xizang Province, Chayu County, Gadui Village, 14–16.V.2015, YPT, Ye Chen, Chao Zhang; 1 ♀, id., but Talin Village, 6–13.VII.2017, Malaise trap, Sang Tuo; 3 ♀, China: Xizang Province, Linzhi City, Bomi County, Shuangyu Village, 8. VIII. 2017, sweeping, Hui-Lin Han; 1 ♀, China: Yunnan Province, Ruili City, Nanjingli Village, 26–27.IV.2013, YPT, Xiang-Xiang Jin, Guo-Hao Zu, Chao Zhang; 1 ♀; id., but Huanglianshan National Nature Reserve, 27–28.VII.2018, YPT, Jun Wu, Ming-Rui Li; 1 ♀, China: Shanxi Province. Ningshan County, Xunyangba Village, 5–6.VIII.2015, YPT, Ye Chen, Chao Zhang; 2 ♀, China, Shandong Province, Qingdao City, Xiaozhushan, 20.V.2014, sweeping, Xiang-Xiang Jin, Guo-Hao Zu, Si-Zhu Liu; 1 ♀, id., but 22–24.V.2014; 1 ♀, China: Heilongjiang Province, Yichun City, Liangshui National Nature Reserve, 30.VI.–2.VII.2018, YPT, Jun Wu, Jun-Jie Fan, Guang-Xin Wang; 1 ♀, id., but 2–3.VII.2018; 1 ♀, China: Guangdong Province, Zhaoqing City, DingHu Mountain, 6–7.V.2019, YPT, Wen-Jian Li, Jun Wu.

##### Diagnosis.

**Figure 7.**
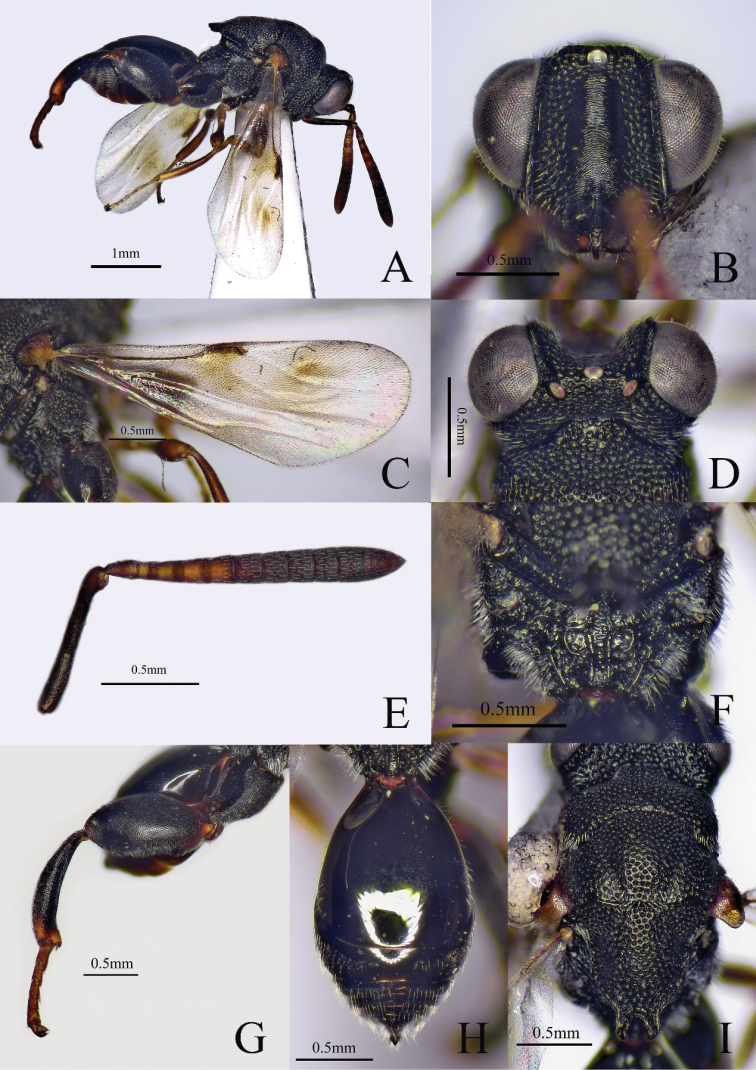
*Haltichellanipponensis*, (female) **A** habitus, lateral view **B** head, front view **C** forewing **D** head, dorsal view **E** antenna **F** propodeum **G** hind leg **H** metasoma **I** mesosoma, dorsal view.

**Female.** Body length 2.7‒4.3 mm, mostly black (Fig. 7A); scape (Fig. 7E) dark brown except brown pedicel to Fu3, eye and ocelli silvery gray (Fig. 7D); tegula brown; fore and mid legs brown, femora slightly darker in middle; hind leg black except yellowish brown tarsus. Head (Fig. 7B, D) 1.1× as wide as long; eye with sparse and short setae; POL 4.9× as long as OOL; scrobe reaching anterior ocellus. Antenna clavate (Fig. 7E), scape a half as long as the remaining antennomeres combined; pedicel longer than Fu1; club coniform, 2× as long as maximum width, 3× as long as and about as broad as the preceding segment; mesoscutellum longer than broad, apex of mesoscutellum (Fig. 7I) with two teeth, distance between outer margins of the two teeth at least 1.5× as long as individual length of teeth. Fore wing largely hyaline (Fig. 7C) with two brown patches; postmarginal vein shorter than stigmal vein; metafemur 2.3× as long as broad (Fig. 7G); metasoma oval (Fig. 7H), 1.6× as long as broad in dorsal view; Gt1 occupying 0.7× length of metasoma.

##### Hosts.

Unknown.

##### Distribution.

China (Heilongjiang [new record], Shanxi [new record], Shandong [new record], Xizang [new record], Yunnan [new record], Guangdong [new record], Taiwan), Japan, India (Narendran 1989), Vietnam (Narendran and van Achterberg 2016).

##### Comments.

Our specimens agree well with the original description except for slight colour differences of the teeth of the mesoscutellum. This is the first record from continental China.

## Supplementary Material

XML Treatment for Haltichella bimaculata

XML Treatment for Haltichella bomiana

XML Treatment for Haltichella strigata

XML Treatment for Haltichella clavicornis

XML Treatment for Haltichella nipponensis
